# Chronic multifocal non-bacterial osteomyelitis in hypophosphatasia mimicking malignancy

**DOI:** 10.1186/1471-2431-7-3

**Published:** 2007-01-23

**Authors:** Hermann J Girschick, Etienne Mornet, Meinrad Beer, Monika Warmuth-Metz, Peter Schneider

**Affiliations:** 1 Children's Hospital, University of Würzburg, Germany; 2 Université de Versailles, Saint Quentin en Yvelines Batiment Fermat Versailles Cedex, France; 3 Dept. of Radiology, Section of Pediatric Radiology, University of Würzburg, Germany; 4 Dept. of Radiology, Section of Neuroradiology, University of Würzburg, Germany; 5 Clinic for Nuclear Medicine, University of Würzburg, Germany

## Abstract

**Background:**

Hypophosphatasia (HP) is characterized by a genetic defect in the tissue-nonspecific alkaline phosphatase (TNSALP) gene and predominantly an autosomal recessive trait. HP patients suffer from reduced bone mineralization. Biochemically, elevated concentrations of substrates of TNSALP, including pyridoxal-5'-phosphate and inorganic pyrophosphate occur in serum, tissues and urine. The latter has been associated with chronic inflammation and hyperprostaglandinism.

**Case presentation:**

We report on 2 affected children presenting with multifocal inflammatory bone lesions mimicking malignancy: A 6 years old girl with short stature had been treated with human growth hormone since 6 months. Then she started to complain about a painful swelling of her left cheek. MRI suggested a malignant bone lesion. Bone biopsy, however, revealed chronic inflammation. A bone scan showed a second rib lesion. Since biopsy was sterile, the descriptive diagnosis of chronic non-bacterial osteomyelitis (CNO) was established. The diagnostic tests related to growth failure were repeated and subsequent analyses demonstrated a molecular defect in the TNSALP gene. The second girl (10 years old) complained about back pain after she had fallen from her bike. X rays of her spine revealed compressions of 2 thoracic vertebrae. At first these were considered trauma related, however a bone scan did show an additional lesion in the right 4^th ^rib. A biopsy of this rib revealed a sterile lympho- plasmocytoid osteomyelitis suggesting multifocal CNO. Further analyses did show a decreased TNSALP in leukocytes and elevated pyridoxal phosphate in plasma, suggesting a heterozygous carrier status of HP.

**Conclusion:**

Chronic bone oedema in adult HP and chronic hyper-prostaglandinism in childhood HP do suggest that in some HP patients bone inflammation is present in conjunction with the metabolic defect. Sterile multifocal osteomyelitis could be demonstrated. Non-steroidal anti-inflammatory treatment achieved complete remission. These cases illustrate chronic inflammation of the bone as a new feature of HP.

## Background

Hypophosphatasia (HP) is characterized by a genetic defect in the gene of the tissue-nonspecific alkaline phosphatase TNSALP [1-3]. There is clinical variability of the HP phenotype: 5 major subtypes (perinatal, infantile, childhood, adult and odontohypophosphatasia) have been described [4]. Biochemical [5] and molecular data [6-9] indicate that the severity of the molecular genetic alterations and subsequently levels of TNSALP are major determinants of the clinical phenotype. Childhood HP presents after the first year of life with rickets causing a short stature, delayed walking and a waddling gait due to bone deformities (genua vara or valga) and chronic skeletal pain. Clinical variability of childhood hypophosphatasia includes short stature, premature loss of teeth, disturbances of gait, muscle weakness, signs of muscle inflammation and chronic pain located mainly in the lower legs [4]. We have previously described the presence of hyperprostaglandinism in these children, which seems to be a sequel of accumulating pyrophosphate crystals especially in soft tissues [10,11]. Non-steroidal anti-inflammatory agents have been effective in treating chronic pain in these children [10]. So far there have been few reports stating that the bone can be inflamed chronically in HP. Therefore, co-presentation of chronic non-bacterial osteomyelitis (CNO, CRMO) and hypophosphatasia had to be considered in our patients. The term CNO is reserved for a clinical entity affecting mainly adolescents. It can have a uni- or multifocal occurrence [12]. Differential diagnosis included several entities including bone malignancies like osteosarcoma, rhabdomyosarcoma, Ewing's sarcoma and histiocytosis [13]. In this regard it is of importance, that multifocal CNO can also cause hyperostosis [12] and osteolytic lesions. Thus, it can be misdiagnosed as malignancy and vice versa [14]. A direct link between HP and sterile osteomyelitis has not been elucidated so far. An inflammatory process secondary to the metabolic defect in HP might be involved [10].

## Case presentation

### Case 1

**Figure 1:**
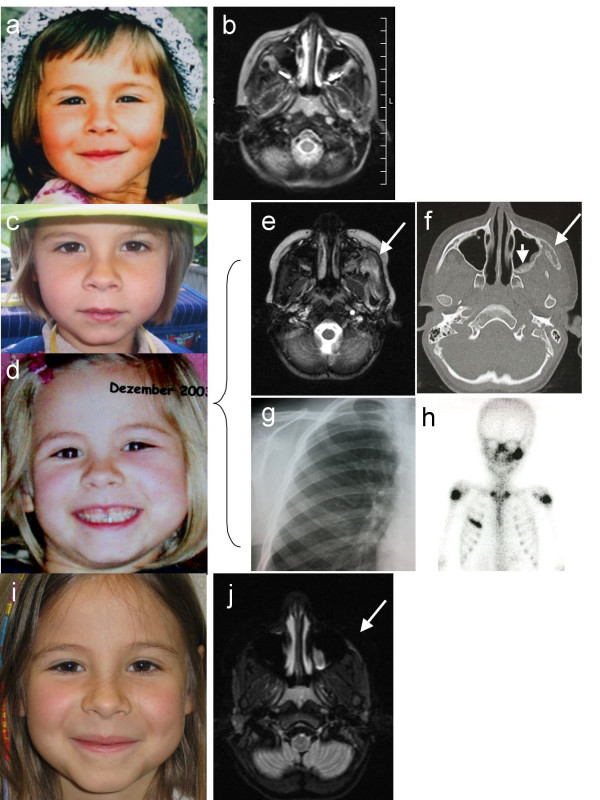
**clinical synopsis of a 6 years old girl with a history of short stature**. Figure 1a shows the patient at the time of suspected growth hormone releasing hormone failure. 2 months after growth hormone therapy started a painful swelling of the cheek was noted (figure 1c) which worsened at the time of presentation 3 months later (figure 1d). Initial magnetic resonance imaging (MRI) was normal (figure 1b: T2-weighted transversal image (slice thickness 5 mm)). Subsequent MRI 5 months later did show a diffuse bone lesion affecting the os zygomaticum and involving surrounding soft tissue (figure 1e: Fat saturated strongly T2-weighted transversal image (slice thickness 5 mm)). Malignancy could not be excluded. Computed tomography revealed hyperostosis in addition to osteolysis (arrows) of the cortical bone (figure 1f). Conventional x-ray of the right hemithorax showed hyperostosis of the anterior 4^th ^rip (figure 1g). Technetium 99m-MDP bone scan showed the involvement of the os zygomaticum and the clinically silent costal lesion (figure 1h). 1 year of non-steroidal anti-inflammatory treatment induced clinical remission (figure 1i). MRI did show a very small signal elevation in the area of the biopsy (figure j: Fat saturated strongly T2-weighted transversal image (slice thickness 5 mm)).

Since birth the child had always been of short stature and low body weight. Growth dynamics showed a constant development of the length along a curve 2 cm below the third percentile. Her weight had increased along the third or slightly below the third percentile during the first six years of life. The genetically determined and proposed length was 1.55 meter (3rd – 10th percentile) (figure 1). A detailed diagnostic workup repetitively revealed a normal concentration of IgF1 (insulin-like growth factor), IgF BP-3 (insulin-like growth factor binding protein). However, stimulation tests using L-arginine and insulin revealed an insufficient rise in human growth hormone (HGH). Therefore a hypothalamic insufficiency was diagnosed. A subsequent cranial MRI was unremarkable (figure 1b). Other reasons for short stature including thyroid insufficiency, cystic fibrosis, chromosomal abnormalities and celiac disease were excluded. Due to a 2 years retardation of the bone age a constitutional retardation of growth also was discussed. Based on two insufficient stimulations of HGH growth hormone therapy was instituted at a standard regimen. Two months later a swelling of the left cheek was noted in addition to local pain while chewing (figure 1c). Dental and ear-nose-throat evaluation were unremarkable. An outpatient antibiotic therapy was started, which did not improve the swelling. Therefore the child was admitted 5 months after HGH treatment had been started (figure 1d). There were no elevated inflammatory parameters (ESR, CRP, leukocytes). A differential blood count did not show signs of malignancy. Further workup revealed normal levels of beta-HCG (human choriogonadotropin), neuron specific enolase and angiotensin converting enzyme. HLA B 27 was absent. MRI showed bone oedema and a diffuse oedematous change of the os zygomaticum surrounding tissue (figure 1e). A cranial computed tomography revealed sclerotic and hyperostotic changes of the os zygomaticum adjacent to the maxillary cavity (figure 1f). The cancellous part and the outer compacta, however, showed osteolysis. Differential diagnosis included bone malignancy, eosinophilic granuloma and chronic inflammation. A technetium bone scan revealed an enhanced uptake in this lesion in addition to another lesion of the right fourth rip (figure 1h). Conventional X-ray of the right hemithorax revealed hyperostosis of the 4^th ^rib (figure 1g). Again malignancy was suspected. To further clarify the diagnosis biopsies were performed including a biopsy of the affected rib and a biopsy of the cheek lesion. Histologically a chronic inflammatory reaction affecting the bone, the periost and the surrounding tissue consisting of predominantly lymphocytes was diagnosed and malignancy was excluded. A detailed microbial workup was performed including eubacteria, mycobacteria and fungi. An universal polymerase chain reaction using primers amplifying ribosomal RNA did not reveal a product [15].

After exclusion of malignancy and an infectious aetiology the descriptive diagnosis of chronic non-bacterial multifocal osteomyelitis (CNO) was made. Since this diagnosis usually affects adolescents other causes were discussed. Despite of an intensive literature research it stayed unclear whether growth hormone treatment can contribute to a chronic inflammatory bone process. During this new re-evaluation relatively low serum levels of previously documented total alkaline phosphatase levels (120, 102, 93, 131 U/l) were noted. Recently, the definition of the lower normal levels (37°C IFCC method) has become difficult to jugde. Compared to a cohort of genetically diagnosed childhood hypophosphatasia patients [16,11] these levels were considered borderline. Therefore we performed a specific measurement of the tissue non-specific alkaline phosphatase in leukocytes, which were below normal (1.2 nmol/min/mg Protein; normal: 2 – 18) [16]. In addition, pyridoxal phosphate in the plasma was significantly elevated (97 ng/ml; normal: 5 – 30 ng/ml). Therefore the diagnosis of a mild form of hypophosphatasia was made. Further genetic diagnostic workup revealed the presence of a heterozygous mutation R374C (c.1171C>T) which was previously described in patients with various forms of hypophosphatasia [8,9]. The mutation is supposed to have a severe effect in view of site-directed mutagenesis experiments and phenotypes of some patients. In this family, the mutation is from maternal origin, since it was found in the mother's genome also. Despite of sequencing the whole coding sequence and intron/exon borders, we did not find any other mutation. In addition we found a number of intronic and exonic polymorphisms (c.330C>T (S93S), c787T>C (Y246H), c.862+20G>T, c862+51G>A, c862+58C>T, c.863-7T>C, c.863-12C>G, c.876A>G(P275P), c.1565T>C(V505A)). Quantitative Multiplex PCR of short fragments of exons 1,3,5,7,9 and 12 did not give evidence of any large deletion in the ALPL gene.

### Case 2

**Figure 2:**
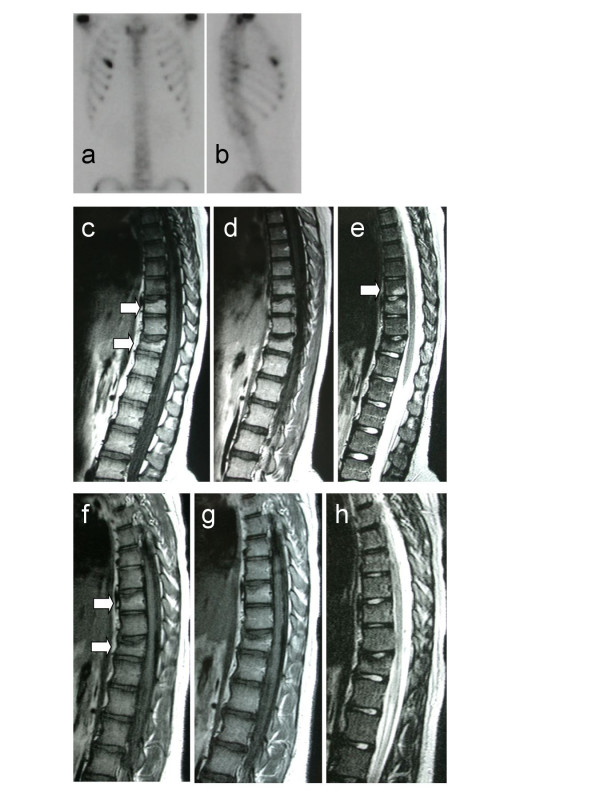
**clinical synopsis of a 10 years old girl with a history of back pain**. Technetium 99m-MDP bone scan (figure 2a and b) shows the rib and multiple osseous spine lesions at diagnosis. Initial magnetic resonance imaging (MRI) revealed a compression of vertebra #9 and 11 (figure 2c: T1-weighted sagittal image (arrows); figure 2d: T1-weighted sagittal image after i.v. gadolinium enhancement), in addition to oedematous changes predominantly in the 8^th ^vertebra (figure 2e: strongly T2-weighted sagittal image; arrow). Subsequent MRI 12 months later no longer showed oedematous changes, however the vertebral compressions were still present (figure 2f: T1-weighted sagittal image (arrows); figure 2g: T1-weighted sagittal image after i.v. gadolinium enhancement; figure 2h: strongly T2-weighted sagittal image).

The second girl (10 years old) complained about back pain after she had fallen from her bike. She had grown normal along the 50th height percentile. X rays of her spine revealed oedematous changes of 3 and compressions of 2 thoracic vertebrae (# 9, 11). At first these were considered trauma related, however, especially posterior oedematous changes of the 8th vertebra were considered not to be typical sequels of a trauma (figure figure 2c–e). In addition, a technetium bone scan did show an additional lesion in the right 4^th ^rib (figure 2a,b). The rib lesion was also analysed by MRI of the thorax. It did show a strong T2/TIRM/STIR signal. Thus Langerhans' cell histiocytosis was suspected at that time. A biopsy of this rib, however, revealed a sterile lympho- plasmocytoid osteomyelitis suggesting multifocal CNO. There were no elevated inflammatory parameters (ESR, CRP, leukocytes). A differential blood count did not show signs of malignancy. Further workup revealed normal serum levels of beta-HCG (human choriogonadotropin), neuron specific enolase, cortisol and angiotensin converting enzyme. HLA B 27 was present. Total alkaline phosphatase levels (401, 426, 316, 378, 185, 189 U/l) measured over time were in the normal range. Compared to a cohort of genetically diagnosed childhood hypophosphatasia patients [16,11] these levels were considered unremarkable. However, a nephelometric measurement of the bone alkaline phosphatase revealed a reduced level (8.9 microg/l; normal 36–80 microg/l). Therefore, a more reliable and specific measurement of the tissue non-specific alkaline phosphatase in leukocytes was done. The result was below normal (1.8 nmol/min/mg Protein; normal: 2 – 18) [16]. In addition, pyridoxal phosphate in the plasma was slightly elevated (31 ng/ml; normal: 5 – 30 ng/ml), phosphoethanolamine excretion in the urine was normal. We suggested that a partial defect in the TNSALP gene was present. She may be a carrier of one severely mutated allele of alkaline phosphatase, because slight elevation of plasma pyridoxal levels has been demonstrated in obligatory heterozygotes (parents of index patients) with severe alleles [16]. Thus, the patient might be a carrier for the hypophosphatasia trait. The family refused a diagnostic genetic workup.

Based on our experience in treating hypophosphatasia patients with non-steroidal anti-inflammatory agents [10,11], in addition to the anti-inflammatory treatment of chronic non-bacterial osteomyelitis patients [12] we started a therapy using naproxen at a dose of 15 mg/kg/d in both patients. This therapeutic protocol was approved by the ethics committee of the University of Wuerzburg.

#### Follow up of patient 1

The treatment rapidly reduced clinical symptoms including pain and soft tissue swelling (figure 1i). Parallel to this clinical improvement subsequent MRI analysis after 3, 6 and 12 months revealed subsequent reduction of bone marrow oedema and local gadolinium enhancement. 12 months later MRI showed almost unremarkable lesions (figure 1j). Mild hyperostosis of the os zygomaticum was still present after 12 months at treatment cessation. 24 months later the patient was still in remission.

#### Follow up of patient 2

The medication did abrogate pain completely after the first 3 months of treatment. Parallel to this clinical improvement subsequent MRI analysis (figure 2c–e) after 3, 6 and 12 (figure 2f–h) months revealed subsequent reduction of bone marrow oedema and local gadolinium enhancement. 12 months later MRI showed an unremarkable 8^th ^thoracic vertebra, whereas the 9th and 11th still showed compression without sign of acute inflammation (figure 2f–h). The rib lesion was unremarkable after 6 months of treatment. No further structural improvement was noted after 24 months. Thus, treatment was terminated.

## Conclusion

These two case reports illustrate several clinical problems. At first chronic inflammatory bone lesions can mimic bone tumors, soft tissue tumors and haematological malignancies including osteosarcoma, rhabdomyosarcoma and Langerhans-cell-histocytosis [15]. Diagnostic imaging including MRI and bone scan might not be sufficient enough to make a proper diagnosis [14]. Findings in diagnostic imaging in the two patients were not different in extent and structure from a cohort of patients with chronic osteomyelitis in whom hypophosphatasia had been excluded [12,14]. Therefore the diagnosis often has to be biopsy proven. However, the quality of the biopsy depends significantly on whether representative areas of the lesion are included. Secondly, microbial workup has to be as detailed and as extensive as possible to exclude rare chronic bacterial osteomyelitis and to avoid unnecessary long-term antibiotic treatment [15]. Thirdly, it shows a dilemma in the care of patients with mild hypophosphatasia or hypophosphatasia traitors. Borderline or slightly reduced alkaline phosphatase values might be overlooked in clinical practice especially because the lower reference range of alkaline phosphatase activity is not defined precisely. In addition, hyperpostaglandinism, which has been demonstrated in hypophosphatasia, might contribute to chronic bone inflammation in this disease [10,11]. It is also unclear whether growth hormone therapy might also have played a role in the first patient's inflammatory process. In addition, hypophosphatasia patients do show changes in their bone structure, which can resemble vitamin D resistant rickets or osteomalacia clinically and on x-rays [4]. Hypophosphatasia patients are prone to bone fractures, but bone inflammation has not been reported in conjunction with osteomalacia or fractures in hypophosphatasia so far. In our cases, even though we can not exclude a contribution of the altered bone structure, inflammatory histologic changes seem to be of a different, supposedly metabolic, nature than structural bone insufficiency alone. Furthermore, minimal and unrecognized trauma might be a trigger factor for the lesions seen in our patients. In healthy individuals one would not expected that bone inflammation might result from such a trauma. However, bone in hypophosphatasia might react differently on the basis of the primary TNSALP and secondary metabolic condition (pyrophosphate, prostaglandins) [11]. A structurally altered rib cage like pigeon-chest is present in some hypophosphatasia patients and might lead to a repetitive minimal trauma at the costochondral junction. Chest structure, however, was unremarkable in our patients. In addition, since a solitary major rib lesion was present in each of our patients a traumatic origin of the lesion seems to be unlikely, because several adjacent lesions could be expected then. Of note, even though the clavicle is affected in CNO predominantly, rib lesions are rare in our and other CNO cohorts [17,12-22].

Even though the precise aetiology of the hypophosphatasia patient's chronic bone inflammation is not known, symptomatic anti-inflammatory treatment was the treatment of choice and was effective.

## Competing interests

The author(s) declare that they have no competing interests.

## Authors' contributions

All authors have contributed to the clinical care of the patients, except EM, who was responsible for the genetic testing. HG was leading the clinical care and treatment strategies. MB, PS and MW-M have performed the diagnostic imaging. All authors have read and approved the final manuscript.

## Pre-publication history

The pre-publication history for this paper can be accessed here:
